# Recalculation of the budgetary impact of risperidone use for Autism Spectrum Disorder: a case study using measured demand data, Brazil, 2017-2019

**DOI:** 10.1590/S2237-96222025v34e20240732.en

**Published:** 2025-08-08

**Authors:** Luis Phillipe Nagem Lopes, Augusto César Sousa Raimundo, Alexander Itria, Rosângela Caetano, Luciane Cruz Lopes

**Affiliations:** 1 Universidade de Sorocaba, Programa de Pós-Graduação em Ciências Farmacêuticas, Sorocaba, SP, Brazil; 2 Universidade do Estado do Rio de Janeiro, Instituto de Medicina Social, Rio de Janeiro, RJ, Brazil; 3 Universidade Estadual de Campinas, Faculdade de Odontologia de Piracicaba, Piracicaba, SP, Brazil; 4 Universidade Federal de São Carlos, Departamento de Gerontologia, São Carlos, SP, Brazil

**Keywords:** Technology Assessment, Biomedical, Analysis of Budgetary Impact, Autism Spectrum Disorder, Risperidone, Case Studies, Evaluación de la Tecnología Biomédica, Análisis de Impacto Presupuestario, Trastorno del Espectro Autista, Risperidona, Estudios de Caso

## Abstract

**Objective:**

To analyze the budgetary impact of use of risperidone for autism spectrum disorder (ASD) in the Brazilian National Health System (SUS).

**Methods:**

This is a case study with a document-based approach, which compared the estimates of the budgetary impact analyses presented in the recommendation reports of the National Commission for the Incorporation of Technologies (CONITEC) for the use of risperidone for ASD with amounts ​​recalculated from measured demand data. The recalculation for children (0-17 years) and adults (≥18 years) was made using data from the Open Health Intelligence Room platform on the dispensing of risperidone in the SUS, considering a three-year time period (2017-2019).

**Results:**

The total budgetary impact over three years of use of risperidone for ASD showed differences between measured demand (children: BRL 10,389,702.70; adults: BRL 15,075,767.80) and that estimated by CONITEC in its recommendation reports (children: R$ 6,579,809.00; adults: R$ 9,877,790.18).

**Conclusion:**

The budgetary impact of use of risperidone for ASD, based on measured demand, differed from the impact initially predicted in CONITEC’s recommendation reports.

## Resumen

**Objetivo:**

Analizar el impacto presupuestario del uso de risperidona para el trastorno del espectro autista (TEA) en el Sistema Único de Salud (SUS).

**Métodos:**

Se trata de un estudio de caso con enfoque documental, que comparó las estimaciones de los análisis de impacto presupuestario presentados en los informes de recomendaciones de la Comisión Nacional para la Incorporación de Tecnologías (Conitec) para el uso de risperidona en TEA con los valores recalculados a partir de los datos de demanda medida. El recálculo se realizó para niños (0-17 años) y adultos (≥18 años) utilizando datos de la plataforma Sala de Inteligencia en Salud Abierta sobre la dispensación de risperidona en el SUS, considerando un horizonte temporal de tres años (2017-2019).

**Resultados:**

El impacto presupuestario total a lo largo de tres años para el uso de risperidona en TEA presentó diferencias entre la demanda medida (niños: R$ 10.389.702,70; adultos: R$ 15.075.767,80) y la estimada por Conitec en los informes de recomendaciones (niños: R$ 6.579.809,00; adultos: R$ 9.877.790,18).

**Conclusión:**

El impacto presupuestario del uso de risperidona en TEA, basado en la demanda medida, fue diferente del impacto inicialmente previsto en los informes de recomendaciones de Conitec.

**Ethical aspects**

This research used public domain anonymized databases.:

## Introduction

The resilience of a health system involves, among other aspects, the ability to adapt and transform itself to improve its functioning in the face of adverse conditions, making it better prepared to face present and future challenges (1). One of the challenges posed to all systems, including the chronically underfunded Brazilian National Health System (*Sistema Único de Saúde* - SUS), is its financial sustainability. This is constantly stressed by the evolution of new (and old) health needs and by the innovation process, which produces a constant flow of new, increasingly costly technologies (2).

Budgetary impact analyses are essential tools for assisting health service managers in making decisions about incorporation of technologies (3). When performed properly, they can estimate the financial consequences of the adoption and diffusion of new technology (3). The way these budgetary impact analyses are conducted is critically evaluated, including with regard to their initial accuracy in relation to the real world (4-7).

In Brazil, important changes have occurred since the creation by the Ministry of Health of its Department of Science and Technology in the 2000s and the Commission for the Incorporation of Technologies (8). Based on Federal Law No. 12401/2011, the National Commission for the Incorporation of Technologies into the Brazilian National Health System (*Comissão Nacional de Incorporação de Tecnologias no Sistema Único de Saúde* - CONITEC) was established (9). This Commission has advised the Ministry of Health on decisions about the incorporation and exclusion of new drugs, products and medical procedures (8). As part of the CONITEC evaluation process provided for in the legal regulations, evaluations of scientific evidence regarding new technologies and economic evaluations are conducted (8). In the scope of economic studies, budgetary impact analyses have been constantly used, not always in association with complete economic evaluations (10).

It is known that many CONITEC evaluations for the incorporation of medical technologies and procedures are performed, but there is little discussion (and practice) regarding disinvestment (11). This is defined as the process of withdrawing (totally or partially) resources from health interventions with low efficiency, that is, those that offer little or no health gains in relation to their cost (11).

Given the high costs that the SUS must bear to ensure universal coverage and the fact that many technologies are incorporated while they are still at a low diffusion stage, the data available for a more precise analysis of the cost-effectiveness relationship are usually scarce (12). Regarding the coverage to be provided by the SUS, measured demand data are generally not available during the incorporation process and can only be evaluated after some time of actual use of the technology (10).

In an ideal scenario, all technologies incorporated should undergo a process of reevaluation of both the scientific evidence of their safety, efficacy and effectiveness, as well as the economic evidence, in order to verify whether the real-world results achieved are in line with those expected (11). Although they are the target of resistance, reevaluation initiatives have been growing worldwide to build more resilient and sustainable health systems (13).

In Brazil, an important milestone in this regard was the development of the methodological guideline for evaluating the performance of health technologies (14). This guideline was published in 2017 by CONITEC in collaboration with other institutions and proposed the performance evaluation flow of technologies incorporated into the SUS, to examine whether the results achieved were in line with those predicted during their incorporation (14). 

In parallel with the publication of the guideline, in 2021, CONITEC, in partnership with the National Council for Scientific and Technological Development (*Conselho Nacional de Desenvolvimento Científico e Tecnológico*), launched a call for proposals for the reevaluation of technologies already incorporated. The call for proposals provided for systematic reviews and budgetary impact analyses for prioritized technologies. Among these, risperidone, a second-generation antipsychotic used in the treatment of aggressive behavior associated with Autism Spectrum Disorder – ASD (15), stood out. Reevaluation of risperidone is justified, since this technology was incorporated more than 10 years ago and belongs to the Specialized Component of Pharmaceutical Assistance (*Componente Especializado da Assistência Farmacêutica*) (16). Although it has a relatively low unit cost, its continued use can represent a significant economic burden for the SUS (15).

The objective of this study was to analyze the budgetary impact of the use of risperidone for ASD in the SUS.

## Methods

### Design

This case study involved a document-based evaluation of the budgetary impact analyses contained in the recommendation reports produced by CONITEC on the incorporation of risperidone into the SUS for use in cases of ASD. The budgetary impact of risperidone was recalculated based on measured demand data for the period 2017-2019.

### Setting

The context of this study was the process of incorporating health technologies into the SUS, based on CONITEC advice (8). CONITEC publishes recommendation reports containing analyses of scientific evidence and economic evaluations (8). The object of this study is the analysis of the budgetary impact of risperidone used for ASD. This medication belongs to the Specialized Component of Pharmaceutical Assistance and its being dispensed requires a Clinical Protocol and Therapeutic Guidelines (15).

### Data source

Two main data sources were used: i) CONITEC recommendation reports for incorporating risperidone into the SUS, publicly available (17,18); ii) retrospective data on measured demand for risperidone in ASD cases extracted from the Open Health Intelligence Room platform (*Sala Aberta de Inteligência em Saúde*). This platform retrieves data from the SUS Information Technology Department, structuring information on medication dispensing in tabulated files (19).

Data on medications forming part of the Specialized Component of Pharmaceutical Assistance, including risperidone, came from the Outpatient Information System and from the National Registry of Health Establishments, administered by the Health Ministry’s Specialized Health Care Secretariat (19). 

### Participants

All individuals registered on the Open Health Intelligence Room platform with confirmed diagnosis of ASD and who received risperidone in the period 2017-2019 were taken into account in order to perform the recalculation (Supplementary Table 1). These individuals were identified by age group, considering children (0-17 years) and adults (≥18 years), and the following codes from the International Statistical Classification of Diseases and Related Health Problems (ICD-10), as established in the Clinical Protocol and Therapeutic Guidelines for the treatment of aggressive behavior associated with ASD: F84.0 (childhood autism), F84.1 (atypical autism), F84.3 (other childhood disintegrative disorder), F84.5 (Asperger syndrome), and F84.8 (other pervasive developmental disorders).

**Table 1:** Parameters adopted in the CONITEC recommendation reports for conducting analysis of the budgetary impact of risperidone use for Autism Spectrum Disorder. Brazil, 2017-2019

Variables	Estimated value (average)	Optimistic (minimum)	Conservative (maximum)	References
Autism Spectrum Disorder prevalence	1.1%	0.3%	2.0%	(20)
Aggressiveness (18-29 years)	31.3%	18.1%	44.4%	(24)
Aggressiveness (30-74 years)	20.0%	9.0%	31.0%	Not identified
Refractoriness	33.5%	22.0%	45.0%	(38)
Hyperprolactinemia incidence	44.9%	37.0%	52.0%	(31)
Risperidone dose (mg)	3	1	6	(40)
Prolactin level (per year)	2	1	4	(41)

Regarding the Analysis of the Budgetary Impact of risperidone for ASD, the population was extracted from CONITEC’ recommendation reports, which are based exclusively on epidemiological data, such as ASD prevalence rates and incidence of aggressiveness (Table 1).

### Variables

The following variables were collected from the recommendation reports: i) date of evaluation and incorporation; ii) age range used in the Budgetary Impact Analysis estimate; iii) population estimate and risperidone expenditure after its incorporation; iv) expenditure on monitoring adverse events; and v) sensitivity and scenario analyses. Data were collected from the Open Health Intelligence Room platform on the total amount of risperidone dispensed annually and the number of people treated by age group during the study period.

### Bias control

The data obtained from the Open Health Intelligence Room platform were compared with those recorded on the Outpatient Information System in order to verify the coherence of the information. Tabulation of the data extracted from the recommendation reports and recalculation based on measured demand were performed independently by two authors, ensuring reproducibility and minimizing errors.

### Study **size**

The study population used in the recalculation was defined based on the total number of people served by the SUS and registered on the Open Health Intelligence Room platform, which guaranteed a representative sample of the measured demand for risperidone used for ASD.

### Data pairing

The data from the CONITEC reports were compared with the data extracted from the Open Health Intelligence Room platform. The recalculation methodology followed the same approach used in the recommendation reports, replicating the proposed model and adjusting the estimates based on measured demand and the unit cost of the technology and laboratory procedures, when necessary (Supplementary Table 2). For recalculation, the population identified on the Open Health Intelligence Room platform was multiplied by the annual cost of acquiring risperidone and by the cost of the adverse event monitoring tests. The cost of risperidone oral solution was considered for children; while for adults the cost of risperidone in tablets was used. SUS laboratory tests were considered for 70.0% of the population served, in accordance with the recommendation reports (17,18).

**Table 2:** Budgetary impact of use of risperidone via the Brazilian National Health System for children and adolescents (0-17 years) with Autism Spectrum Disorder and differences between estimates by epidemiological demand and estimates based on measured demand. Brazil, 2017-2019

Year	Budgetary impact - epidemiological demand (BRL)	Budgetary impact - measured demand (BRL)	Difference (BRL)^a^
**Risperidone purchasing**			
2017	744,782.59	1,647,132.00	902,349.41
2018	1,489,565.18	3,106,676.64	1,617,111.46
2019	1,986,086.91	5,109,624.96	3,123,538.05
Total	4,220,434.68	9,863,433.60	5,642,998.92
**Laboratory tests for control of use**			
2017	584,559.56	139,774.81	-444,784.75
2018	853,742.30	146,130.96	-707,611.34
2019	921,072.46	240,363.33	-680,709.13
Total	2,359,374.32	526,269.10	-1,833,105.22

^a^Negative amounts indicate overestimation of the model calculated by epidemiological demand.

### Statistical **methods**

Descriptive statistics were used to compare the budgetary impact data of the population estimated in the CONITEC recommendation reports and those of measured demand. The data were analyzed using Microsoft Excel 2016. In order to ensure the reliability of the results, tabulation and checking were carried out independently by two researchers.

Regarding the statistical analyses performed in the recommendation reports, sensitivity analyses were considered whenever available, including optimistic and conservative values ​​(Table 1). These analyses were considered in order to verify whether the measured demand estimates fell within the ranges considered by the recommendation report after the sensitivity analyses.

### Data access and cleaning methods

The data used are publicly available and were extracted from official sources. Data cleaning included checking consistency between the different systems used, removing duplicates and manually reviewing outliers before final analysis.

## Results

The total budgetary impact estimated in the CONITEC reports for three years of risperidone use in the treatment of children and adolescents with ASD via the SUS was BRL 4,220,434.68 for the acquisition of the drug and BRL 2,359,374.32 for adverse event monitoring tests. These expenditures were estimated based on the expected population of 43,199 children and adolescents. The population served by the SUS was 19,160 children and adolescents (Supplementary Table 1). The budgetary impact over three years was BRL 9,863,433.60 for the acquisition of risperidone and BRL 526,269.10 for adverse event monitoring tests (Table 2). This represented a difference of BRL 5.7 million more in spending on risperidone and BRL 1.8 million less in spending on adverse event monitoring tests, compared to the CONITEC report estimates (Table 2).

According to CONITEC estimates, the annual cost per child and adolescent with ASD using risperidone would be BRL 152.31. After analyzing measured demand, we found that the actual cost per child and adolescent was BRL 542.26, which represented an increase of BRL 389.69 in relation to the estimated amount.

For the adult population, the budgetary impact estimated in the CONITEC report was BRL 4,543,080.85 for risperidone purchasing and BRL 5,334,709.33 for adverse event monitoring tests, with the expectation of serving 97,622 adults over the three-year time period. During the period analyzed, 185,132 individuals aged 18 or older received care via the SUS (Supplementary Table 2), resulting in a budgetary impact of BRL 9,851,376.60 for risperidone and BRL 5,224,391.24 for adverse event monitoring tests (Table 3). This represented a difference of BRL 5.3 million more in spending on risperidone and a reduction of BRL 110,318.09 in costs for adverse event monitoring tests, compared with the CONITEC report estimates (Table 3).

**Table 3:** Budgetary impact of use of risperidone via the Brazilian National Health System for adults (≥18 years) with Autism Spectrum Disorder and differences between estimates by epidemiological demand and estimates based on measured demand. Brazil, 2017-2019

Year	Budgetary impact epidemiological demand (BRL)	Budgetary impact measured demand (BRL)	Difference (BRL)^a^
**Risperidone purchasing**			
2017	801,720.15	824,009.40	22,289.25
2018	1,603,440.30	4,091,832.00	2,488,391.70
2019	2,137,920.40	4,935,535.20	2,797,614.80
Total	4,543,080.85	9,851,376.60	5,308,295.75
**Laboratory tests for control of use**			
2017	1,327,524.10	1,659,030.62	331,506.52
2018	1,925,739.12	1,616,073.06	-309,666.06
2019	2,081,446.11	1,949,287.56	-132,158.55
Total	5,334,709.33	5,224,391.24	-110,318.09

^a^Negative amounts indicate overestimation of the model calculated by epidemiological demand.

According to CONITEC estimates, the annual cost per adult with ASD would be BRL 101.18. After the recalculation, we found that the cost was BRL 81.43 per adult, which represented a difference of BRL 19.75 less than the CONITEC estimate.

When comparing the recalculation with the amounts ​​presented in the recommendation report for the adult population, the findings differed from the average amount estimated by CONITEC and from the confidence intervals calculated with the conservative and optimistic scenarios (Supplementary Table 3).

## Discussion

This case study showed important differences between the estimates of the budgetary impact analyses presented in the CONITEC recommendation reports, based only on epidemiological data, and the expenditures estimated from measured SUS demand. The limitations of the epidemiological data for estimating the SUS demand and the budgetary impact became evident. These differences corroborate the Analysis of Budgetary Impact carried out previously, which investigated the budgetary impact of risperidone for ASD with a five-year projection (2022-2026). Although the analysis did not use retrospective consumption data, but rather projections, substantial differences were also found between the use of the epidemiological perspective and measured demand (6).

It should be noted that the technology underwent unit price changes not foreseen in the CONITEC reports, such as Ordinance No. 3,789/2017, which changed the unit price of risperidone 1 mg to BRL 0.10 and the price of the oral solution vial to BRL 21.41. These changes in the acquisition cost generated significant differences in the recalculation, since the change in the unit acquisition cost implies an increase in annual expenditure on risperidone. This may directly affect the total estimated amounts ​​for treatment per person served.

Regarding population delimitation, the CONITEC recommendation report used the estimated prevalence of ASD from a study conducted in England (20). Despite the remarkable robustness of those data, with the stratified approach based on a probabilistic sample and considerable statistical power, they come from evidence external to the Brazilian population that may present differences, including significant ones, given that known prevalence is related, among other factors, to diagnostic capacity (20). Although the increase in ASD prevalence is a global reality, divergences could be observed between countries due to social and economic factors and the availability of adequate and valid instruments for the identification of this clinical condition (21).

ASD is a highly heterogeneous and underdiagnosed clinical condition (21). Diagnosis itself is influenced by socioeconomic aspects and access to health services (22). Since population surveys are extremely scarce in Brazil for several clinical conditions in the field of mental health and, when available, may be inaccurate, measured demand can be an important strategy for budgetary impact analyses. It is expected that, in relation to ASD, this reality will progressively improve thanks to Law No. 13861/2019, which made it mandatory to include data related to this population in demographic censuses (23).

In the CONITEC recommendation report, incidence of aggressiveness among individuals with ASD aged 18 to 29 years was measured from a convenience sample of 76 adult participants with ASD from a province in the far west of Canada (24). The measure of aggressiveness used was self-reported by the research participants, and there was no validated measure for identifying aggressive behavior; this applies to the reference used in the report on the incidence of aggressiveness in individuals over 29 years of age (24).

Aggressive behavior associated with ASD is still a difficult construct to define and quantify, because it involves different factors. The individual’s own reality and surroundings can influence the level of aggressiveness and, inevitably, its measurement method (25). The imprecision of these measurements and the limitations inherent to the clinical picture of this population make it difficult to define the specific budgetary impact analysis population when epidemiological demand is used exclusively. They may also represent differences, perhaps significant, in relation to the national reality (26).

Limitations of this study are those inherent to the measured demand data taken from the SUS information system that were used to perform the recalculation. These data do not accurately estimate the barriers related to access and social inequalities that affect use of medications, including second-generation antipsychotics (27). Based on the possibilities of measured demand and epidemiological demand in budgetary impact analyses, it became clear that these estimates are not reality, but a simulation of it, with weaknesses and methodological limitations, as with any mathematical model (28).

In relation to Brazil, it should be noted that, in some cases, measured demand or drug consumption data will only be available several years after the technology has been incorporated into the SUS. This emphasizes the importance of constant reevaluations and a systematic process of reinvestment or disinvestment in health technologies. Use of drug dispensing data for measured demand in the context of economic evaluations should be encouraged in the reevaluation of health technologies already incorporated into the SUS.

Analysis of expenditures on monitoring adverse events through laboratory tests is an aspect that deserves considerable attention when it comes to second-generation antipsychotics. These medications cause adverse events in at least 50% of the population with ASD, leading to their discontinuation (29). Prescribing antipsychotic medications for use by children and young people increases mortality, which highlights the need for great rigor in prescribing and monitoring adverse events (30).

In the case analyzed, the study used to measure the percentage of individuals who develop hyperprolactinemia related to use of risperidone – a critical adverse event related to this medication, caused by the increase in prolactin levels and which can cause gynecomastia – was conducted with a population of Thai individuals, which also used a convenience sample (31). Although those data come from a real-world study, they have low external validity and may not represent Brazilian reality.

Adverse effects of second-generation antipsychotics are little documented, especially with regard to ASD. Safety data for these drugs are generally extrapolated from evidence from short-term randomized clinical trials, which range from 6 to 24 weeks (32). With regard to the limitation present in the Analysis of Budgetary Impact contained in the CONITEC recommendation report regarding adverse event monitoring tests, not even measured demand data can fill this gap.

The Analysis of Budgetary Impact that examined expenditures on risperidone and monitoring of adverse events and compared epidemiological demand and measured demand reported the limitations of SUS administrative data that correlate risperidone dispensing data with laboratory tests for monitoring adverse events (4). It is currently unfeasible to quantify and monitor adverse effects in this population based on SUS administrative data; as such, it becomes impossible to obtain the epidemiological and clinical profile of these SUS service users.

In the United States, Johnson & Johnson compensated risperidone users with up to US$8 billion due to gynecomastia, which can occur due to increased prolactin levels associated with risperidone use (33). Gynecomastia can be prevented by constantly monitoring prolactin levels, as described in clinical protocols and international guidelines (34). Lack of knowledge about the profile and monitoring of these medications in the SUS, as well as failure to monitor this population longitudinally using robust prospective cohorts, can pose risks to this population, especially pediatric patients, since gynecomastia requires surgical procedures to achieve remission. This, combined with other important monitoring procedures related to metabolism, such as triglyceride, cholesterol and blood glucose levels, can increase the risk of mortality (35).

Substantial discrepancies between epidemiological demand and measured demand have been pointed to previously (4-6). Many budgetary impact analyses, despite strictly following methodological guidelines, may remain inaccurate in relation to real-world data when epidemiological data are scarce (36). 

In addition to the problems related to the management of the SUS during the COVID-19 pandemic, the System has other long-standing problems that hinder its resilience (37). Among these “chronic” problems of the SUS is the weakness of administrative databases, which, in addition to assisting in management, are also important tools for situational analysis of the services and technologies offered by the SUS. If these databases are not strengthened, both the sustainability of the SUS and also evaluation and monitoring are impossible. If budgetary impact analyses are not progressively strengthened, which inevitably depend on overcoming underfunding and training human resources, it will be more complex to predict, more accurately, the economic consequences of incorporating technologies and their long-term monitoring.

This case study showed that, regardless of the very well-established mathematical models used in CONITEC’s recommendation reports, in the absence of reliable epidemiological data that represent Brazilian reality, budgetary impact analyses may remain imprecise and incompatible with reality. In the case analyzed, the budgetary impact of use of risperidone for ASD, based on measured demand, differed from the impact initially predicted in CONITEC’s recommendation reports. Technology reevaluations are necessary in order to monitor the performance of medical technologies incorporated into the SUS, aiming at greater sustainability of the System and more efficient allocation of resources.

## Data Availability

All the data are available in the Results section and in the Supplementary Material.
