# Supplementary material for: Recalculation of the budgetary impact of risperidone use for Autism Spectrum Disorder: a case study using measured demand data, Brazil, 2017-2019
Source: Epidemiol Serv Saude. 2025 Aug 8;34:e20240732. doi: 10.1590/S2237-96222025v34e20240732.en (PMC12342727; doi:10.1590/S2237-96222025v34e20240732.en)
Supplement: Supplementary file 1 [file 2237-9622-ress-34-e20240732-supp01.pdf]

Tabela Suplementar 1 – Estimativa da população atendida com Transtorno do Espectro Autista em uso de risperidona. Brasil, 2017-2019

Ano	Adultos	Crianças
2017	37.626	3.170
2018	66.860	6.046
2019	80.646	9.944
Total	185.132	19.160

Tabela suplementar 2 - Parâmetros adotados para o recálculo do impacto orçamentário a partir da demanda aferida do uso de risperidona para o Transtorno do Espectro Autista. Brasil, 2017-2019.

Exames laboratoriais e tecnologia por paciente	Ano 1	Ano 2	Ano 3	**Referências**
Glicemia e perfil lipídico (R$)	42,69	42,69	14,23	SIGTAP
Dosagem de prolactina (R$)	20,30	20,30	20,30	SIGTAP
Risperidona comprimidos (R$)	21,90	61,20	61,20	SIGTAP
Risperidona solução oral (R$)	519,60	513,84	513,84	SIGTAP, 2016; SIGTAP, 2017

Tabela Suplementar 3 – Análises de sensibilidade do impacto orçamentário da risperidona no Transtorno do Espectro Autista para a população adulta e comparação com os dados de demanda aferida. Brasil, 2017-2019

**Ano**	**Demanda epidemiológica (R$)**	**Intervalos de confiança 95% (R$)**	**Demanda aferida (R$)**
2017	3.486.283,66	3.363.228,09 a 3.609.339,22	2.773.213,04
2018	3.793.587,26	3.653.996,66 a 3.933.177,86	3.025.060,64
2019	4.507.176,40	4.341.971,32 a 4.672.381,49	3.648.804,08
