# Supplementary material for: Recalculation of the budgetary impact of risperidone use for Autism Spectrum Disorder: a case study using measured demand data, Brazil, 2017-2019
Source: Epidemiol Serv Saude. 2025 Aug 8;34:e20240732. doi: 10.1590/S2237-96222025v34e20240732.en (PMC12342727; doi:10.1590/S2237-96222025v34e20240732.en)
Supplement: Translation

# Translation: Recálculo do impacto orçamentário da risperidona para uso no transtorno do espectro autista: estudo de caso com dados de demanda aferida, Brasil, 2017-2019

**DOI:** 10.1590/S2237-96222025v34e20240732.pt

**Authors:** Luis Phillipe Nagem Lopes, Augusto César Sousa Raimundo, Alexander Itria, Rosângela Caetano, Luciane Cruz Lopes

**Affiliations:** 1 Universidade de Sorocaba, Programa de Pós-Graduação em Ciências Farmacêuticas, Sorocaba, SP, Brasil; 2 Universidade do Estado do Rio de Janeiro, Instituto de Medicina Social, Rio de Janeiro, RJ, Brasil; 3 Universidade Estadual de Campinas, Faculdade de Odontologia de Piracicaba, Piracicaba, SP, Brasil; 4 Universidade Federal de São Carlos, Departamento de Gerontologia, São Carlos, SP, Brasil

**Keywords:** Avaliação de Tecnologias em Saúde, Análise de Impacto Orçamentário, Transtorno do Espectro Autista, Risperidona, Estudo de Caso

## Abstract

**Objetivo:**

Analisar o impacto orçamentário do uso de risperidona para o transtorno do espectro autista (TEA) no Sistema Único de Saúde (SUS).

**Métodos:**

Trata-se de estudo de caso com abordagem documental, que comparou as estimativas das análises de impacto orçamentário apresentadas nos relatórios de recomendação da Comissão Nacional de Incorporação de Tecnologias (Conitec) para o uso da risperidona no TEA com os valores recalculados a partir de dados de demanda aferida. Fez-se o recálculo para crianças (0-17 anos) e adultos (≥18 anos) por meio dos dados da Sala Aberta de Inteligência em Saúde de dispensação de risperidona no SUS, considerando o horizonte temporal de três anos (2017-2019).

**Resultados:**

O impacto orçamentário total em três anos para uso de risperidona no TEA apresentou diferenças entre a demanda aferida (crianças: R$ 10.389.702,70; adultos: R$ 15.075.767,80) e o estimado pela Conitec nos relatórios de recomendação (crianças: R$ 6.579.809,00; adultos: R$ 9.877.790,18).

**Conclusão:**

O impacto orçamentário do uso da risperidona no TEA, a partir da demanda aferida, diferiu do impacto previsto inicialmente nos relatórios de recomendação da Conitec.

**Aspectos éticos**

Esta pesquisa utilizou bancos de dados de domínio público e anonimizados.:

## Introdução

A resiliência de um sistema de saúde envolve, entre outros aspectos, a capacidade de se adaptar e se transformar para melhorar seu funcionamento diante de condições adversas, tornando-o mais preparado para enfrentar desafios presentes e futuros (1). Um dos desafios colocados a todos os sistemas, inclusive ao Sistema Único de Saúde (SUS), cronicamente subfinanciado, se refere à sua sustentabilidade financeira. Esta é estressada constantemente pelo evoluir de novas (e antigas) necessidades de saúde e pelo processo de inovação que produz constante fluxo de novas tecnologias cada vez mais custosas (2).

As análises de impacto orçamentário são ferramentas essenciais para auxiliar gestores na tomada de decisão sobre a incorporação de tecnologias (3). Quando realizadas adequadamente, podem estimar as consequências financeiras da adoção e difusão de nova tecnologia (3). A condução dessas análises de impacto orçamentário é avaliada criticamente, inclusive no que diz respeito à sua acurácia inicial em relação ao mundo real (4-7). 

No Brasil, importantes mudanças têm surgido desde a criação do Departamento de Ciência e Tecnologia nos anos 2000 e da Comissão de Incorporação de Tecnologias, vinculados ao Ministério da Saúde (8). A partir da Lei Federal nº 12.401/2011, a Comissão Nacional de Incorporação de Tecnologias no Sistema Único de Saúde (Conitec) foi instituída (9). Tal comissão tem assessorado o Ministério da Saúde nas decisões sobre incorporação e exclusão de novos medicamentos, produtos e procedimentos médicos (8). Como parte do processo avaliativo da Conitec previsto no regramento legal, são conduzidas avaliações das evidências científicas acerca das novas tecnologias e avaliações econômicas (8). No escopo dos estudos econômicos, as análises de impacto orçamentário têm sido constantemente utilizadas, nem sempre em associação com avaliações econômicas completas (10). 

Sabe-se que muitas avaliações da Conitec para a incorporação de tecnologias e procedimentos médicos são realizadas, mas pouco se discute (e se pratica) o desinvestimento (11). Este é definido como o processo de retirada (total ou parcial) de recursos de intervenções em saúde com baixa eficiência, isto é, que ofereçam pouco ou nenhum ganho em saúde mediante ao seu custo (11).

Diante dos altos custos que o SUS precisa arcar para garantir a cobertura universal e do fato de que muitas tecnologias são incorporadas ainda em estágio de baixa difusão, os dados disponíveis para a análise mais precisa da relação custo-efetividade costumam ser escassos (12). Tratando-se da cobertura a ser atendida no SUS, os dados de demanda aferida geralmente não estão disponíveis durante a incorporação e só podem ser avaliados após algum tempo de uso propriamente dito da tecnologia (10).

No cenário ideal, todas as tecnologias incorporadas deveriam passar pelo processo de reavaliação tanto das evidências científicas de sua segurança, eficácia e efetividade, quanto das evidências econômicas, a fim de verificar se os resultados de mundo real alcançados estão de acordo com aqueles esperados (11). Embora seja alvo de resistência, iniciativas de reavaliação têm crescido no mundo para a construção de sistemas de saúde mais resilientes e sustentáveis (13). 

No Brasil, um marco importante nesse aspecto foi a elaboração da diretriz metodológica para avaliação de desempenho de tecnologias em saúde (14). Essa diretriz foi publicada em 2017 pela Conitec em colaboração com outras instituições e propôs o fluxo de avaliação de desempenho das tecnologias incorporadas no SUS, para examinar se os resultados alcançados estavam em consonância com os previstos durante a incorporação (14). 

Paralelamente à publicação da diretriz, em 2021 a Conitec, em parceria com o Conselho Nacional de Desenvolvimento Científico e Tecnológico, lançou a chamada em edital para a reavaliação de tecnologias já incorporadas. O edital previa a realização de revisões sistemáticas e análises de impacto orçamentário para tecnologias priorizadas. Entre estas, destacou-se a risperidona, um antipsicótico de segunda geração utilizado no tratamento do comportamento agressivo associado ao transtorno do espectro autista – TEA (15). A reavaliação da risperidona é justificável, pois essa tecnologia foi incorporada há mais de 10 anos e pertence ao Componente Especializado da Assistência Farmacêutica (16). Embora tenha valor unitário relativamente baixo, seu uso contínuo pode representar carga econômica significativa para o SUS (15).

O objetivo deste estudo é analisar o impacto orçamentário do uso de risperidona para o TEA no SUS.

## Métodos

### Delineamento do estudo

Tratou-se de estudo de caso com base na avaliação documental das análises de impacto orçamentário presentes nos relatórios de recomendações realizados pela Conitec na incorporação da risperidona no SUS para uso no TEA. Fez o recálculo do impacto orçamentário da risperidona a partir dos dados de demanda aferida no período 2017-2019. 

### Contexto

O contexto deste estudo foi o processo de incorporação de tecnologias em saúde no SUS que é assessorada pela Conitec (8). Esta publica relatórios de recomendação contendo análises das evidências científicas e avaliações econômicas (8). As análises de impacto orçamentário da risperidona para o TEA são o objeto deste estudo. Esse medicamento pertence ao Componente Especializado da Assistência Farmacêutica e sua dispensação requer Protocolo Clínico e Diretrizes Terapêuticas (15). 

### Fonte dos dados

Foram utilizadas duas principais fontes de dados: i) relatórios de recomendação da Conitec para a incorporação da risperidona no SUS, disponíveis publicamente (17,18); ii) dados retrospectivos de demanda aferida da risperidona no TEA extraídos da plataforma Sala Aberta de Inteligência em Saúde. Essa plataforma recupera dados do Departamento de Informática do SUS, estruturando informações de dispensação de medicamentos em arquivos tabulados (19).

Os dados sobre medicamentos do Componente Especializado da Assistência Farmacêutica, incluindo a risperidona, foram provenientes do Sistema de Informação Ambulatorial e do Cadastro Nacional de Estabelecimentos de Saúde, administrados pela Secretaria de Atenção Especializada à Saúde (19). 

### Participantes

Para o recálculo, foram consideradas todas as pessoas cadastradas na Sala Aberta de Inteligência em Saúde com diagnóstico confirmado de TEA e que receberam risperidona no período 2017-2019 (Tabela suplementar 1). A identificação dessas pessoas foi realizada por meio da faixa etária, considerando crianças (0-17 anos) e adultos (≥18 anos), e dos seguintes códigos da Classificação Estatística Internacional de Doenças e Problemas Relacionados à Saúde (CID-10), conforme estabelecido no Protocolo Clínico e Diretrizes Terapêuticas para o tratamento do comportamento agressivo associado ao TEA: F84.0 (autismo infantil), F84.1 (autismo atípico), F84.3 (outro transtorno desintegrativo da infância), F84.5 (síndrome de Asperger) e F84.8 (outros transtornos globais do desenvolvimento).

Em relação à análise de impacto orçamentário da risperidona para o TEA, a população foi extraída dos relatórios de recomendação da Conitec, que utilizou exclusivamente dados epidemiológicos, como taxas de prevalência do TEA e incidência de agressividade (Tabela 1). 

**Tabela 1:** Parâmetros adotados nos relatórios de recomendação da Conitec para a condução da análise de impacto orçamentário da risperidona para o Transtorno do Espectro Autista. Brasil, 2017-2019

Variáveis	Valor estimado (média)	Otimista (mínimo)	Conservador (máximo)	Referências
Prevalência do Transtorno do Espectro Autista	1,1%	0,3%	2%	(20)
Agressividade (18 a 29 anos)	31,3%	18,1%	44,4%	(24)
Agressividade (30 a 74 anos)	20,0%	9,0%	31,0%	Não identificada
Refratariedade	33,5%	22,0%	45,0%	(38)
Taxa de absorção anual média	60,0%	30,0%	80,0%	(39)
Incidência de hiperprolactinemia	44,9%	37,0%	52,0%	(31)
Dose de risperidona (mg)	3	1	6	(40)
Dosagem de prolactina (por ano)	2	1	4	(41)

### Variáveis

As seguintes variáveis foram coletadas dos relatórios de recomendação: i) data da avaliação e incorporação; ii) faixa etária utilizada na estimativa da análise de impacto orçamentário; iii) estimativa populacional e de gastos com risperidona após incorporação; iv) gastos com monitoramento de eventos adversos; e v) análises de sensibilidade e de cenário. Na plataforma Sala Aberta de Inteligência em Saúde para a realização do recálculo, foram coletados dados sobre o total de risperidona dispensada anualmente e o número de pessoas atendidas por faixa etária no período do estudo.

### Controle de viés

Os dados obtidos na plataforma Sala Aberta de Inteligência em Saúde foram comparados com aqueles registrados no Sistema de Informação Ambulatorial para verificar a coerência das informações. A tabulação dos dados extraídos dos relatórios de recomendação e a implementação do recálculo com base na demanda aferida foram realizadas de forma independente por dois autores, garantindo reprodutibilidade e minimizando erros.

### Tamanho do estudo

A população do estudo no recálculo foi definida com base no total de pessoas atendidas pelo SUS e registrados na plataforma Sala Aberta de Inteligência em Saúde, o que garantiu a amostra representativa da demanda aferida para risperidona no TEA.

### Pareamento dos dados

Os dados dos relatórios da Conitec foram comparados com os dados extraídos da plataforma Sala Aberta de Inteligência em Saúde. A metodologia de recálculo seguiu a mesma abordagem utilizada nos relatórios de recomendação, replicando o modelo proposto e ajustando as estimativas, com base na demanda aferida, e o valor unitário da tecnologia e dos procedimentos laboratoriais, quando necessário (Tabela suplementar 2). Para o recálculo, multiplicou-se a população identificada na Sala Aberta de Inteligência em Saúde pelo custo anual de aquisição de risperidona e pelo custo dos exames de monitoramento de eventos adversos. Foi considerado o custo da risperidona em solução oral para as crianças; para os adultos, utilizou-se o custo da risperidona em comprimidos. Os exames laboratoriais no SUS foram considerados para 70,0% da população atendida, conforme os relatórios de recomendação (17,18).

### Métodos estatísticos

Utilizaram-se estatísticas descritivas para comparar os dados de impacto orçamentário da população estimada nos relatórios de recomendação da Conitec e os da demanda aferida. Os dados foram analisados no Microsoft Excel 2016. Para garantir a fidedignidade dos resultados, a tabulação e a conferência foram realizadas independentemente por dois pesquisadores. 

Em relação às análises estatísticas realizadas nos relatórios de recomendação, consideraram-se as análises de sensibilidade sempre que disponíveis, incluindo os valores otimistas e conservadores (Tabela 1). Essas análises foram consideradas a fim de verificar se as estimativas da demanda aferida estavam contidas nos intervalos considerados pelo relatório de recomendação após as análises de sensibilidade.

### Acesso aos dados e métodos de limpeza

Os dados utilizados são de acesso público e foram extraídos de fontes oficiais. A limpeza dos dados incluiu verificação de consistência entre os diferentes sistemas utilizados, remoção de duplicatas e revisão manual dos valores discrepantes antes da análise final.

## Resultados

O impacto orçamentário total estimado nos relatórios da Conitec para três anos de uso da risperidona no tratamento de crianças e adolescentes com TEA no SUS foi R$ 4.220.434,68 para a aquisição do medicamento e R$ 2.359.374,32 para os exames de monitoramento de eventos adversos. Esses gastos foram previstos com base na população esperada de 43.199 crianças e adolescentes. No SUS, a população atendida foi 19.160 crianças e adolescentes (Tabela suplementar 1). O impacto orçamentário em três anos foi R$ 9.863.433,60 para a aquisição de risperidona e R$ 526.269,10 para exames de monitoramento de eventos adversos (Tabela 2). Isso representou a diferença de R$ 5,7 milhões a mais em gastos com risperidona e R$ 1,8 milhão a menos com exames de monitoramento de eventos adversos, em comparação com as estimativas do relatório da Conitec (Tabela 2).

**Tabela 2:** Impacto orçamentário do uso de risperidona no Sistema Único de Saúde para crianças e adolescentes (0 a 17 anos) com Transtorno do Espectro Autista e diferenças entre as estimativas por demanda epidemiológica e baseada em demanda aferida. Brasil, 2017-2019

Ano	Impacto orçamentário demanda epidemiológica (R$)	Impacto orçamentário demanda aferida (R$)	Diferença (R$)^a^
**Aquisição da risperidona**			
2017	744.782,59	1.647.132,00	902.349,41
2018	1.489.565,18	3.106.676,64	1.617.111,46
2019	1.986.086,91	5.109.624,96	3.123.538,05
Total	4.220.434,68	9.863.433,60	5.642.998,92
**Exames laboratoriais de controle do uso**			
2017	584.559,56	139.774,81	-444.784,75
2018	853.742,30	146.130,96	-707.611,34
2019	921.072,46	240.363,33	-680.709,13
Total	2.359.374,32	526.269,10	-1.833.105,22

^a^ Valores negativos indicam superestimação do modelo calculado pela demanda epidemiológica.

De acordo com as estimativas da Conitec, o custo anual por criança e adolescente com TEA em uso de risperidona seria R$ 152,31. Após a análise da demanda aferida, observou-se que o gasto real por criança e adolescente foi R$ 542,^26^, o que representou o acréscimo de R$ 389,69 em relação ao valor estimado.

Para a população adulta, o impacto orçamentário estimado no relatório da Conitec foi R$ 4.543.080,85 para a aquisição da risperidona e R$ 5.334.709,33 para exames de monitoramento de eventos adversos, com a previsão de atender 97.622 adultos no horizonte temporal de três anos. No período analisado, 185.132 pessoas com 18 anos ou mais foram atendidas no SUS (Tabela suplementar 2), o que resultou no impacto orçamentário de R$ 9.851.376,60 para a risperidona e R$ 5.224.391,24 para exames de monitoramento de eventos adversos (Tabela 3). Isso representou a diferença de R$ 5,3 milhões a mais em gastos com risperidona e a redução de R$ 110.318,09 nos custos com exames de monitoramento de eventos adversos, em comparação com as estimativas do relatório da Conitec (Tabela 3).

**Tabela 3:** Impacto orçamentário do uso de risperidona no Sistema Único de Saúde para adultos (a partir de 18 anos) no Transtorno do Espectro Autista e diferenças entre as estimativas por demanda epidemiológica e baseada em demanda aferida. Brasil, 2017-2019

Ano	Impacto orçamentário demanda epidemiológica (R$)	Impacto orçamentário demanda aferida (R$)	Diferença (R$)^a^
**Aquisição da risperidona**			
2017	801.720,15	824.009,40	22.289,25
2018	1.603.440,30	4.091.832,00	2.488.391,70
2019	2.137.920,40	4.935.535,20	2.797.614,80
Total	4.543.080,85	9.851.376,60	5.308.295,75
**Exames laboratoriais de controle do uso**			
2017	1.327.524,10	1.659.030,62	331.506,52
2018	1.925.739,12	1.616.073,06	-309.666,06
2019	2.081.446,11	1.949.287,56	-132.158,55
Total	5.334.709,33	5.224.391,24	-110.318,09

^a^ Valores negativos indicam superestimação do modelo calculado pela demanda epidemiológica.

Conforme as estimativas da Conitec, o custo anual por adulto com TEA seria R$ 101,18. Após o recálculo, verificou-se que o gasto foi R$ 81,43 por adulto, o que representou a diferença de R$ 19,75 a menos em relação à estimativa anterior.

Ao comparar o recálculo com os valores apresentados no relatório de recomendação para a população adulta, os achados diferiram do valor médio estimado pela Conitec e dos intervalos de confiança calculados com os cenários conservadores e otimistas (Tabela suplementar 3).

## Discussão

Este estudo de caso mostrou importantes diferenças entre as estimativas das análises de impacto orçamentário apresentadas nos relatórios de recomendação da Conitec, com base apenas em dados epidemiológicos, e os gastos estimados a partir da demanda aferida no SUS. Ficaram evidentes as limitações dos dados epidemiológicos para estimar a demanda do SUS e o impacto orçamentário. Essas diferenças corroboram a análise de impacto orçamentário realizada anteriormente, a qual investigou o impacto orçamentário da risperidona para o TEA com projeção de cinco anos (2022-2026). Apesar de a análise não ter utilizado dados retrospectivos de consumo, mas projeções, diferenças substanciais também foram encontradas entre o uso da perspectiva epidemiológica e a demanda aferida (6).

Destaca-se que a tecnologia passou por mudanças no preço unitário não previstas nos relatórios da Conitec, a exemplo da Portaria nº 3.789/2017, que alterou o valor unitário da risperidona 1 mg para R$ 0,10 e do frasco de solução oral para R$ 21,41. Essas modificações no custo de aquisição geraram importantes diferenças no recálculo, uma vez que a alteração no custo unitário de aquisição implica o aumento do gasto anual com a risperidona. Isso pode afetar diretamente os valores totais estimados para o tratamento por pessoas atendidas.

Acerca da delimitação populacional, o relatório de recomendação da Conitec utilizou a estimativa de prevalência do TEA de um estudo realizado na Inglaterra (20). Apesar de notável robustez desses dados, com a abordagem estratificada com base na amostra probabilística e no considerável poder estatístico, eles são provenientes da evidência externa à população brasileira que podem apresentar diferenças, inclusive, significativas, dado que a prevalência conhecida está relacionada, entre outros fatores, à capacidade diagnóstica (20). Apesar de o aumento da prevalência do TEA ser uma realidade mundial, divergências puderam ser observadas entre os países devido aos fatores sociais e econômicos e à disponibilidade de instrumentos adequados e válidos para a identificação desta condição clínica (21).

O TEA é uma condição clínica bastante heterogênea e subdiagnosticada (21). O próprio diagnóstico é influenciado por aspectos socioeconômicos e de acesso a serviços de saúde (22). Uma vez que os inquéritos populacionais são extremamente escassos no Brasil para diversas condições clínicas no campo da saúde mental e, quando disponíveis, podem ser pouco precisos, a demanda aferida pode ser importante estratégia para análises de impacto orçamentário. Espera-se que, em relação ao TEA, essa realidade melhore progressivamente com a Lei nº 13.861/2019, que passou a obrigar a inclusão de dados relacionados a essa população nos censos demográficos (23).

No relatório de recomendação da Conitec, a incidência de agressividade entre indivíduos com TEA entre 18 e 29 anos foi aferida a partir da amostra de conveniência, envolvendo 76 participantes adultos com TEA de determinada província do extremo oeste do Canadá (24). A medida de agressividade utilizada foi o próprio relato dos participantes da pesquisa, não havendo nenhuma medida validada para a identificação do comportamento agressivo; isso se aplica à referência utilizada no relatório sobre a incidência de agressividade em indivíduos maiores de 29 anos (24). 

O comportamento agressivo associado ao TEA ainda é construto de difícil definição e quantificação, porque envolve diferentes fatores. A própria realidade do indivíduo e seu entorno podem influenciar no nível de agressividade e, fatalmente, em sua forma de mensuração (25). A imprecisão dessas medidas e as limitações inerentes ao quadro clínico dessa população dificulta a especificidade da delimitação populacional das análises de impacto orçamentário quando se utiliza exclusivamente a demanda epidemiológica. Também podem representar diferenças, talvez significativas, para a realidade nacional (26). 

Consideram-se as limitações inerentes aos dados de demanda aferida retirados do sistema de informação do SUS que foram utilizados no recálculo deste estudo. Esses dados não dão conta de estimar com precisão as barreiras relacionadas ao acesso e as desigualdades sociais que atravessam o uso de medicamentos, inclusive dos antipsicóticos de segunda geração (27). Com base nas possibilidades de demanda aferida e epidemiológica em análises de impacto orçamentário, foi explícito que essas estimativas não são a realidade, mas a simulação desta, com fragilidades e limitações metodológicas, como todo modelo matemático (28). 

Tratando-se do Brasil, salienta-se que, em alguns casos, os dados de demanda aferida, ou de consumo de medicamentos, só estarão disponíveis depois de alguns anos de incorporação da tecnologia no SUS. Isso fomenta a importância de constantes reavaliações e processo sistemático de reinvestimento ou desinvestimento de tecnologias em saúde. O uso de dados de dispensação de medicamentos para a demanda aferida no contexto das avaliações econômicas deve ser encorajado na reavaliação de tecnologias de saúde já incorporadas no SUS.

A análise de gastos com monitoramento de eventos adversos por meio dos exames laboratoriais é um aspecto que merece bastante atenção tratando-se dos antipsicóticos de segunda geração. Esses medicamentos provocam eventos adversos em, pelo menos, 50,0% da população com TEA, levando à sua descontinuação (29). A prescrição de medicamentos antipsicóticos para crianças e jovens aumenta a mortalidade, o que ressalta a necessidade de grande rigor na prescrição e no monitoramento dos eventos adversos (30). 

No caso analisado, o estudo utilizado para mensurar o percentual de indivíduos que desenvolvem hiperprolactinemia relacionada ao uso de risperidona – evento adverso crítico relacionado a esse medicamento, provocado pelo aumento da dosagem de prolactina e que pode provocar a ginecomastia – foi realizado em uma população de indivíduos tailandeses, cuja amostra também foi de conveniência (31). Apesar de esses dados serem provenientes de um estudo de mundo real, possuem baixa validade externa e podem não representar a realidade brasileira. 

Efeitos adversos dos antipsicóticos de segunda geração são pouco documentados, principalmente no TEA. Os dados de segurança desses medicamentos, em geral, são extrapolados das evidências provenientes de ensaios clínicos randomizados de curto tempo de seguimento, que varia entre 6 e 24 semanas (32). Tratando-se da limitação presente na análise de impacto orçamentário do relatório de recomendação da Conitec referente aos exames de monitoramento de eventos adversos, nem mesmo os dados de demanda aferida conseguem suprir essa lacuna. 

A análise de impacto orçamentário que examinou os gastos com risperidona e o monitoramento dos eventos adversos e que comparou a demanda epidemiológica e a aferida relatou as limitações dos dados administrativos do SUS que correlacionem os dados de dispensação da risperidona aos exames laboratoriais para monitoramento de eventos adversos (4). É inviável na atualidade a quantificação e o monitoramento de efeitos adversos nessa população a partir dos dados administrativos do SUS; portanto, o perfil epidemiológico e clínico dos usuários torna-se impossibilitado.

Nos Estados Unidos, a Johnson & Johnson indenizou usuários de risperidona em até 8 bilhões de dólares devido à ginecomastia, que pode ocorrer devido ao aumento da dosagem de prolactina, associado ao uso da risperidona (33). A ginecomastia pode ser prevenida com o monitoramento constante da dosagem de prolactina, conforme descrito nos protocolos clínicos e nas diretrizes internacionais (34). Desconhecer o perfil e o monitoramento desses medicamentos no SUS, bem como não acompanhar de forma longitudinal essa população a partir de robustas coortes prospectivas, pode trazer riscos à população, principalmente pediátrica, uma vez que a ginecomastia demanda procedimentos cirúrgicos para a remissão. Isso somado a outros monitoramentos importantes relacionados ao metabolismo, como dosagem de triglicerídeos, colesterol e glicemia, podem aumentar o risco de mortalidade (35). 

Discrepâncias substanciais já foram sugeridas entre as medidas de demandas epidemiológica e aferida (4-6). Muitas análises de impacto orçamentário, apesar de seguirem irrestritamente diretrizes metodológicas, podem permanecer imprecisas em relação aos dados de mundo real, quando os dados epidemiológicos são escassos (36). 

Além dos problemas relacionados à gestão do SUS durante a pandemia da covid-19, existem outros problemas, já antigos no sistema, que dificultam a sua resiliência (37). Entre esses problemas “crônicos” do SUS, inclui-se a fragilidade dos bancos de dados administrativos que, ao mesmo tempo que auxiliam na gestão, também são importantes ferramentas de análise situacional dos serviços e das tecnologias ofertadas no SUS. Sem o fortalecimento dos bancos de dados, impossibilitam-se a sustentabilidade do SUS, a avaliação e o monitoramento. Sem o fortalecimento progressivo das análises de impacto orçamentário, que depende inevitavelmente da superação do desfinanciamento e da formação de recursos humanos, será mais complexo prever, de forma mais precisa, as consequências econômicas da incorporação de tecnologias e seu monitoramento a longo prazo. 

Este estudo de caso mostrou que, independentemente dos modelos matemáticos muito bem consolidados que são utilizados nos relatórios de recomendação da Conitec, na ausência de dados epidemiológicos confiáveis e que representem a realidade brasileira, as análises de impacto orçamentário podem permanecer imprecisas e incompatíveis com a realidade. No caso analisado, o impacto orçamentário do uso da risperidona no TEA, a partir da demanda aferida, diferiu do impacto previsto inicialmente nos relatórios de recomendação da Conitec. As reavaliações de tecnologias são necessárias para o monitoramento do desempenho das tecnologias médicas incorporadas no SUS, visando à maior sustentabilidade do sistema e à alocação mais eficiente dos recursos.

## Data Availability

Todos os dados estão disponíveis na seção Resultados e no material suplementar.
